# Paravertebral Well-Differentiated Liposarcoma with Low-Grade Osteosarcomatous Component: Case Report with 11-Year Follow-Up, Radiological, Pathological, and Genetic Data, and Literature Review

**DOI:** 10.1155/2017/2346316

**Published:** 2017-03-09

**Authors:** Nicolas Macagno, Stéphane Fuentes, Gonzague de Pinieux, André Maues de Paula, Sébastien Salas, Jean-Camille Mattéi, Charlotte Dupuis, Romain Appay, Alain Aurias, Henry Dufour, Dominique Figarella-Branger, Corinne Bouvier

**Affiliations:** ^1^Department of Pathology, APHM, Marseille, France; ^2^Department of Neurosurgery, APHM, Marseille, France; ^3^Department of Pathology, University Hospital, Tours, France; ^4^Department of Oncology, APHM, Marseille, France; ^5^Department of Orthopedic Surgery, APHM, Marseille, France; ^6^Department of Molecular Biology, Curie Institute, Paris, France

## Abstract

Despite being one of the most frequent soft-tissue sarcomas, well-differentiated liposarcoma has never been reported near the spine. The authors present the case of a 67-year-old man with progressive history of back pain. Physical examination revealed a mass located within the right paravertebral muscles. MR and CT imaging showed a heavily ossified central mass surrounded by a peripheral fatty component. No connection with the underlying bone was detected on imagery and during surgery. After surgical resection, histopathological examination revealed a tumor harboring combined features of well-differentiated liposarcoma and low-grade osteosarcoma. Tumor cells displayed overexpression of MDM2, CDK4, and P16 by immunohistochemistry and CGH revealed amplification of 12q13-15 as the only genetic imbalance. MDM2 FISH analysis was performed but was inconclusive. The pathological, immunohistochemical, and genetic features, the differential diagnoses, and the therapeutic management of this unusual tumor are discussed. No complementary treatment was performed initially. Following first treatment, two recurrences occurred 6 and 9 years later, both displaying histological features similar to the first occurrence. Radiotherapy was started after the second recurrence. Follow-up shows no evidence of disease 11 years after initial diagnosis. This case was unusual due to the paravertebral location of the tumor and its divergent differentiation.

## 1. Introduction

Liposarcoma (LPS) is the most common type of adult soft-tissue sarcoma. The term LPS encompasses a heterogeneous group of neoplasms with different clinicopathological presentation, behavior, and genomic alterations although all share lipogenic differentiation. The WHO classification of soft-tissue tumors recognizes well-differentiated liposarcoma (WDL), dedifferentiated liposarcoma (DL), myxoid/round cell liposarcoma (M/RCL), and pleomorphic liposarcoma (PL). The most frequent sites of involvement of LPS are the retroperitoneum, the deep soft tissues of extremities, and the spermatic cord. Paravertebral involvement by LPS is rare [1] and M/RCL and PL are the most frequent types encountered [2–7]. WDL/DL has been reported near the spine once, but the main mass originated from the mediastinum and spread to the spinal cervical region [8]. In DL, a variety of heterologous differentiation can be present including myo-, osteo-, or chondrogenic. When bone formation occurs in DL, the osteogenic component usually shows high-grade osteosarcomatous features. A rare variant of WDL harboring low-grade mature bone formation has been described. In such cases the term well-differentiated liposarcoma with a low-grade osteosarcomatous component (WDL/LGO) was proposed. Of the 9 cases of WDL/LGO described, the sites of involvement were similar to the conventional sites of involvement of WDL such as the retroperitoneum and deep soft tissue of extremities.

We report an interesting occurrence of a WDL/LGO in a 67-year-old man, unusual by its divergent differentiation and its location within the muscles of the paravertebral location.

## 2. Case Presentation

**Figure 1:**
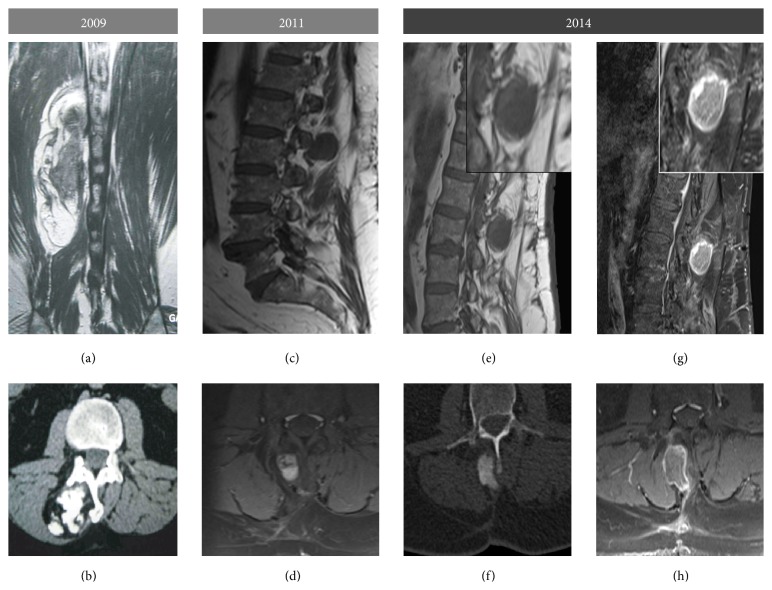
Imaging data of the different occurrences of the tumor: Gadolinium enhanced T1-weighted MR sequence, coronal section (a), Gadolinium enhanced T1-FATSAT weighted MR sequence, parasagittal (g), and axial sections (h), CT scan without injection, axial sections (b), T1-weighted MR sequence, parasagittal sections (c and e), and T1-FATSAT weighted MR sequence, axial section (d and f). There was no obvious radiological connection of the mass with the underlying bone, ruling out a surface tumor. Histological areas corresponding to bone formation were seen as calcified clusters on CT scans, surrounded by a peripheral rim of tumorous fat identified as hypersignal in T1-weighted RM sequences. The two local recurrences showed similar features; however, the ossified core was more homogeneously calcified compared to the first occurrence.

A 67-year-old man with a year-long history of chronic back pain presented with a large lump located at the thoracolumbar junction. CT scan showed an 8 × 4 × 4 cm tumorous mass lying in the paravertebral left muscles and composed of a central hyperdense core and a lower density rim (Figure 1(b)). MR imaging showed that the tumor combined a peripheral fatty component surrounding an ossified core (Figure 1(b)). Surgical resection was performed.

### 2.1. Pathological Findings

**Figure 2:**
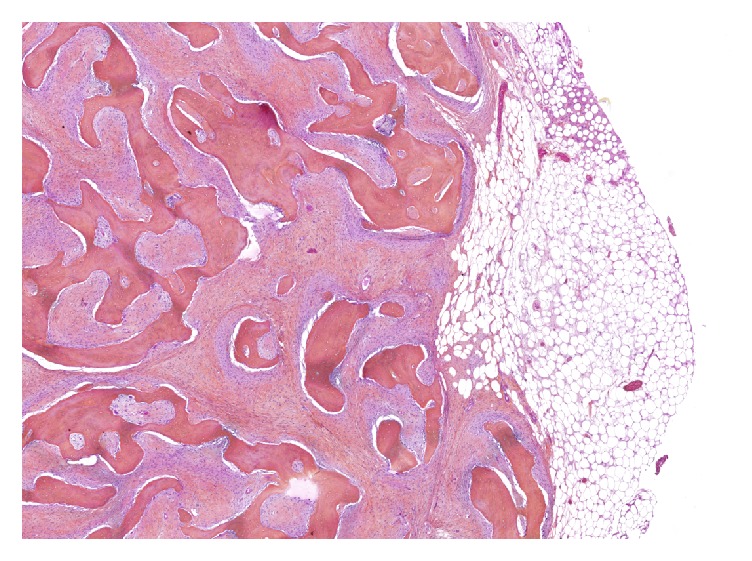
Histological features of the first occurrence: general view of the tumor. A central zone of osteogenesis forms an ossified core intermixed with a low-grade fibroblastic component all of which is surrounded by a mature lipogenic component that extends to the margins of the tumor.

**Figure 3:**
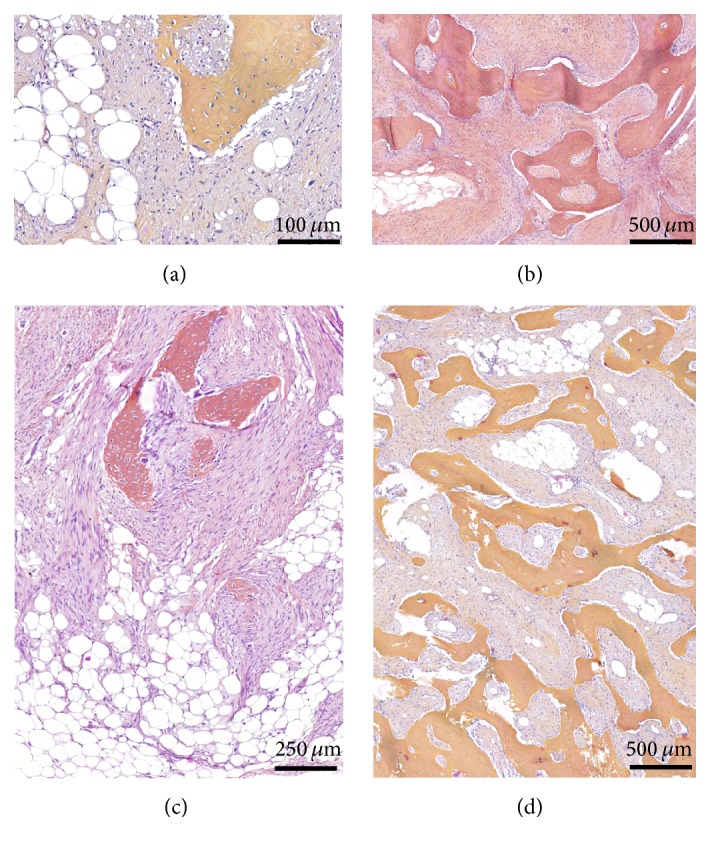
Histological features of the different occurrences of the tumor. (a) Well-differentiated liposarcoma component, first occurrence. Lipogenic clusters are entrapped within a stromal proliferation composed of fusiform cells. A cluster of bone formation is obvious. (b) Low-grade osteosarcoma component, first occurrence. The osteogenic areas are composed of long trabeculae of woven bone associated with the same proliferation of fusiform stromal cells in the intertrabecular spaces. These stromal cells are fusiform, elongated with moderate nuclear atypias. These morphological findings are very similar to low-grade osteosarcoma. (c) and (d) Histological aspects of the recurrences. The 2011 recurrence (c) and the 2014 recurrence (d) showed very similar histological findings compared to the first occurrence of the tumor.

**Figure 4:**
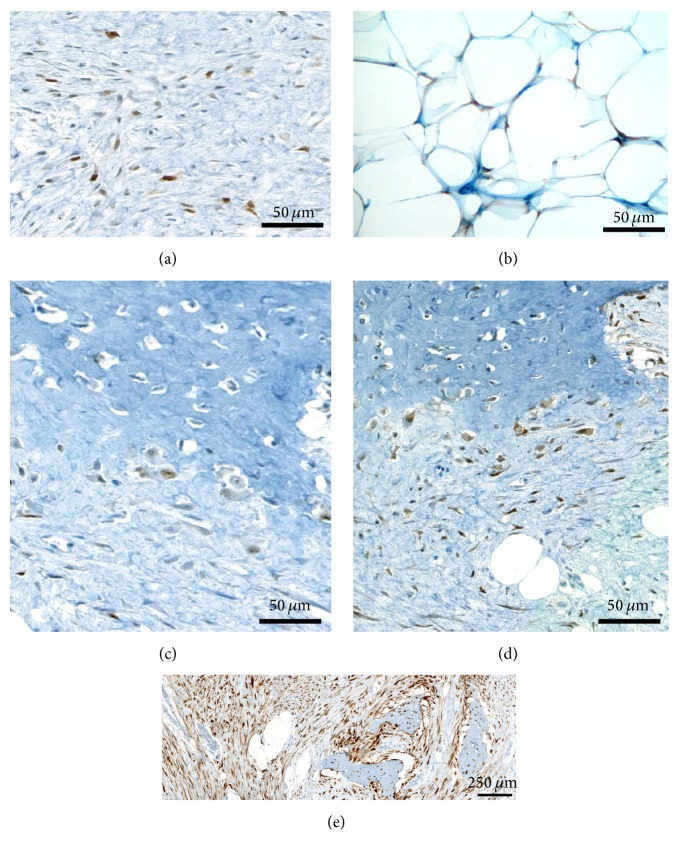
Immunostaining of MDM2, CDK4, and P16: (a), (b), and (c) Immunostaining with anti-MDM2 antibody. MDM2 nuclear positivity is present in 5–10% of stromal cells (a), in some lipoblasts (b), and in rare osteoblasts rimming neoformed bone (c). (d) Immunostaining with anti-CDK4 antibody: more diffuse nuclear but also cytoplasmic staining was present in stromal cells and osteoblasts (20–30%). (e) Immunostaining with anti-P16 antibody: albeit not specific, diffuse and strong positivity with P16 was present in stromal cells, cells rimming or within bone trabeculae and also in lipoblasts.

On gross examination, the mass was composed of a central ossified core surrounded by a yellowish adipose tissue. Microscopically, distinct histological patterns were present (Figure 2): a main component (80%) composed of areas of osteogenesis and a minor peripheral component (20%) of well-differentiated adipose tissue, both of which were intermingled with an abundant fibroblastic stroma. Bone-forming areas were arranged in long, parallel, and narrow trabeculae of woven bone surrounded by a low-grade population of spindle cells similar to what is commonly observed in low-grade osteosarcoma (LGO) (Figure 3(b)). The fatty component was composed of mature anisocytic adipocytes arranged in lobules, with rare spindle cells within their septa that displayed large hyperchromatic nuclei, a morphological finding usually observed in low-grade ALN/WDL (Figure 3(a)). Immunostains showed that the osteogenic, lipogenic, and fibrogenic territories displayed nuclear overexpression of MDM2 (Figures 4(a), 4(b), and 4(c)) and CDK4 (Figure 4(d)), with 5–10% and 20–30% of nuclei stained, respectively. P16 was overexpressed by all tumors cells, with an intense and diffuse pattern of expression (Figure 4(e)). Surgical margins were considered positive since they were in contact with the lipogenic and/or fibrogenic component. This finding was confirmed as immunostains performed on the margins revealed positivity of the cells with MDM2 and P16. FISH analysis of* MDM2* was attempted several times on all occurrences, but in all case hybridization was not conclusive, probably owing to decalcification. Comparative Genomic Hybridization (CGH) revealed amplification of chromosome 12q13-15 as the only significant genomic imbalance, a region where* MDM2* and* CDK4* genes are located. Since there were no radiological nor macroscopic proofs of a connection with the underlying bone, the association of lipogenic and osteogenic differentiation favored the diagnosis of WDL/LGO.

### 2.2. Postoperative Course

No complementary treatment was initially performed. The patient experienced two local recurrences 6 years and 9 years after initial diagnosis; both were surgically resected and local radiotherapy was initiated following the first recurrence. CT and MR imaging of these two recurrences are shown in Figures 1(c)to 1(h). On both of the resected specimens, histological features were identical to the first resection with subtle variation in the proportion of the two components (Figures 3(c) and 3(d)).

## 3. Discussion

We have reported another case of WDL/LGO which was unusual owing to its location in the paraspinal soft tissues.

Histologically, the differential diagnoses of a heavily ossified soft-tissue mass in this particular location may encompass different neoplasms: myositis ossificans, heterotopic ossification (soft-tissue osteomas), ossifying fibromyxoid tumor, or even extraskeletal osteosarcoma. Myositis ossificans typically shows a zonal phenomenon with a fasciitis-like cellular center surrounded by an outer layer of mature lamellar bone. Osteomas are rare well circumscribed lesions composed of mature trabeculae of bone with fatty or hematopoietic marrow. Ossifying fibromyxoid tumor presents as pseudo-encapsulated mass, lobulated by fibrous septa that may display mature bone formation in 80% of cases. Neoplastic cells are arranged in cordonal architecture within a fibromyxoid or hyaline matrix and usually display S100 expression. Albeit very rare, extraskeletal LGO has been reported near the spine. Extraskeletal LGO displays histologic features resembling those of a parosteal osteosarcoma but without adipose component nor connection with the underlying bone [9]. In our cases, the overall architecture, the presence of lipogenic and osteogenic components intermingled with moderately atypical spindle cells, the absence of connection with the underlying bone, and the expression of MDM2/CDK4/P16 ruled out these diagnoses.

Soft-tissue LPS are far more common than extraskeletal LGO. In LPS, low-grade mature bone formation is possible but is a rare phenomenon. Bone formation has been described with or without association with a high-grade dedifferentiated component. Until recently, mature bone formation in LPS was considered metaplastic, but the development of molecular and immunohistochemical techniques to assess the amplification of* MDM2* argued for its neoplastic nature: both the osteogenic and lipogenic components of these tumors displayed amplification of* MDM2*. Low-grade bone formation has also been described in peculiar forms of ALN/WDL which arbor a “meningothelial-like” whorling architecture [10, 11]: most of these cases showed abundant osteogenesis in most of the cases. In these neoplasms, bone formation is directly and intimately associated with the whorls, either at their periphery or directly originating from within them. In all instances, the bone was low-grade consisting of mature trabeculae rimmed by osteoblasts. Low-grade bone formation can also occur without this whorling pattern and has been reported by Yoshida et al. [12] in a series of 9 cases. Yoshida reported that the proportion of LGO ranged from 5 to 50%. In this series, most of the tumors displayed nuclear expression of MDM2 and/or CDK4 similarly to our case and dedifferentiation under the form of a transformation to high-grade osteosarcoma occurred in three cases. In our case, LGO was the main component in the primary and the recurrent tumors and no dedifferentiation occurred.

The prognostic significance of a LGO component in WDL may not be pejorative following the example of heterologous differentiation in DL [13]. In the series of Yoshida et al. none of the patients experienced distant metastases. In our case, the patient experienced an 11-year history free of metastasis in spite of two local recurrences due to incomplete resection. Due to its low-grade aspect, the fatty component may eventually be overlooked and incompletely resected which may lead to local recurrences as our case advocates, and the recognition of the malignant nature of the adipose component is important for clinical management, a feature that pathologists dealing with this rare tumor should be aware of. To help assess its tumoral nature, ancillary techniques such as MDM2/CDK4, HMGA2, or P16 immunohistochemistry can be helpful since all of these markers are frequently overexpressed in ALT/WDL/DL [14–16]. More recently, it has been reported that diffuse and intense P16 staining is highly sensitive for ALT/WDL/DL, although P16 lacks specificity and needs to be combined with MDM2/CDK4 for an accurate diagnosis [17, 18]. FISH can also be performed to confirm the presence of* MDM2* amplification, especially on surgical margin in doubtful cases. For tumors developing in the limbs, resection followed by radiotherapy is recommended to avoid recurrences [19]. In our case radiotherapy was not performed initially because of the spinal medulla vicinity. Close radiologic follow-up was advised and detected two local recurrences treated by surgery. Finally, adjuvant radiotherapy was started after the second recurrence.

Before MDM2 testing was routinely available, other cases of WDL/LGO may have been overlooked: a case of an axillary extraskeletal LGO with a lipomatous mass closely associated to it has been reported in 1991. In this particular case, the fatty component interpreted as lipoma might have represented a low-grade liposarcoma which was underrecognized [20], as MDM2 testing was not routinely available at the time the report was published.

More recently, a parosteal osteoliposarcoma was reported [21], also defined by WDL and LGO coexistence, but arising from the cortex of the underlying bone just like parosteal osteosarcoma, which in our case could not be proven by radiology, gross examination, or histology. Further molecular techniques could not be performed on this parosteal osteoliposarcoma, although the cells of both components were immunoreactive for CDK4.

We have reported a paravertebral WDL/LGO in which both components can be histologically mistaken for a benign process, particularly the fat-forming component. Immunohistochemical and molecular studies may be required for accurate diagnosis. The long-term prognosis seems good depending on adequate local treatment to avoid recurrences.
